# Engineered Removal of PD-1 From the Surface of CD19 CAR-T Cells Results in Increased Activation and Diminished Survival

**DOI:** 10.3389/fmolb.2021.745286

**Published:** 2021-10-13

**Authors:** R. S. Kalinin, V. M. Ukrainskaya, S. P. Chumakov, A. M. Moysenovich, V. M. Tereshchuk, D. V. Volkov, D. S. Pershin, E. G. Maksimov, H. Zhang, M. A. Maschan, Y. P. Rubtsov, A. V. Stepanov

**Affiliations:** ^1^ M.M. Shemyakin and Yu.A. Ovchinnikov Institute of Bioorganic Chemistry of the Russian Academy of Sciences, Moscow, Russia; ^2^ Faculty of Biology, Lomonosov Moscow State University, Moscow, Russia; ^3^ Dmitry Rogachev National Medical Research Center of Pediatric Hematology, Oncology and Immunology, Moscow, Russia; ^4^ State Key Laboratory of Medicinal Chemical Biology and College of Life Sciences, Nankai University, Tianjin, China; ^5^ Department of Chemistry, The Scripps Research Institute, La Jolla, CA, United States

**Keywords:** PD-1, PD-L1, protein expression blockers, tumor microenvironment, CAR T cells

## Abstract

CAR-T cell therapy is the most advanced way to treat therapy resistant hematologic cancers, in particular B cell lymphomas and leukemias, with high efficiency. Donor T cells equipped *ex vivo* with chimeric receptor recognize target tumor cells and kill them using lytic granules. CAR-T cells that recognize CD19 marker of B cells (CD19 CAR-T) are considered the gold standard of CAR-T therapy and are approved by FDA. But in some cases, CD19 CAR-T cell therapy fails due to immune suppressive microenvironment. It is shown that tumor cells upregulate expression of PD-L1 surface molecule that binds and increases level and signal provided by PD-1 receptor on the surface of therapeutic CAR-T cells. Induction of this negative signaling results in functional impairment of cytotoxic program in CAR-T cells. Multiple attempts were made to block PD-1 signaling by reducing binding or surface level of PD-1 in CAR-T cells by various means. In this study we co-expressed CD19-CAR with PD-1-specific VHH domain of anti-PD-1 nanobody to block PD-1/PD-L1 signaling in CD19 CAR-T cells. Unexpectedly, despite increased activation of CAR-T cells with low level of PD-1, these T cells had reduced survival and diminished cytotoxicity. Functional impairment caused by disrupted PD-1 signaling was accompanied by faster maturation and upregulation of exhaustion marker TIGIT in CAR-T cells. We conclude that PD-1 in addition to its direct negative effect on CAR-induced signaling is required for attenuation of strong stimulation leading to cell death and functional exhaustion. These observations suggest that PD-1 downregulation should not be considered as the way to improve the quality of therapeutic CAR-T cells.

## Introduction

Recent advances in the treatment of oncologic disorders are based on the use of monoclonal antibodies that invigorate immune system, and development of adoptive T cell therapies. The most successful example of T cell application in cancer immunotherapy is the CAR-T cell technology. It utilizes the transfer of donor T cells armed with the recombinant Chimeric T cell Receptors (CAR) (Weber et al., 2020). These receptors can bind target molecules on the surface of tumor cells with high specificity and affinity. Interaction between the CAR and its target triggers signaling events inside the CAR-T cells that result in their activation and initiation of cytotoxicity responsible for killing of tumor cells. Unfortunately, the CAR-T cell therapy is effective currently only for the treatment of hematologic malignancies (Lee et al., 2015; Majzner and Mackall, 2019). The major issue that limits practical usefulness of the CAR-T technology is cancer microenvironment which suppresses activation of the CAR-T cells (Gajewski et al., 2013). Often, tumor cells upregulate surface ligands of receptors, also called immune checkpoints (including the CTLA-4/CD80, CD86 and PD-1/PD-L1, PD-L2 ligand/receptor pairs), that normally keep in check excessively activated T cells (Buchbinder and Desai, 2016). PD-1 is a membrane receptor that under steady state conditions is mainly expressed by a small proportion of conventional T cells and by most T regulatory lymphocytes (Francisco et al., 2010). However, upon activation, most CD4^+^ and CD8^+^ conventional T cells augment PD-1 expression (Keir et al., 2008). Results of *in vivo* (Yeku et al., 2017) and clinical (Abiko et al., 2015) studies demonstrate that upon activation, T-cells secrete IFN-γ, which has been shown to dynamically upregulate programmed death ligand-1 (PD-L1) expression on tumors cells (Platanias, 2005; Dorand et al., 2016). Antibody-mediated blockage of these interactions between T cells and tumor cells leads to re-activation of suppressed anti-tumor immunity and subsequent killing of tumor cells (Parry et al., 2005). Several therapeutic mAbs against inhibitory receptors PD-1 (or its ligand) and CTLA-4 are approved for treatment of various types of oncologic disorders (Topalian et al., 2015). Therefore, the idea to block PD-1/PD-L1 interaction to increase the oncolytic potential of therapeutic CAR-T cells is very appealing. There are multiple reports suggesting that combining of the CAR-T cells transfer with administration of mAbs that block the immune checkpoints increases the efficiency of treatment (Brahmer et al., 2010; Topalian et al., 2014; McDermott et al., 2015). Another approach is delivery of a checkpoint’s inhibitors by CAR-T cells expressing soluble PD-1-blocking scFv. *In vivo* studies demonstrated that CAR-T cells that secrete PD-1-blocking scFv enhance survival of mice in syngeneic and preclinical xenogeneic hematologic and solid tumor models (Rafiq et al., 2018). Interruption of PD-1/PD-L1 ligation via CRISPR-mediated deletion of PD-L1 on ovarian cancer significantly improved the efficacy of adoptively transferred second-generation CAR-T cells in preclinical models (Yeku et al., 2017).

On the other side, the attempts to downregulate surface level of PD-1 in the CAR-T cells were made as well by various means. It has been shown that forced withdrawal of PD-1 from the surface of CAR-T cells can be achieved by co-expression of PD-1 -specific VHH fused to ER/Golgi retention peptides. The group of protein expression blockers (PEBLs) allows to colocalize complexes of protein of interest with specific antibody inside the cell and, hence, block its surface expression (Kamiya et al., 2018; Kamiya et al., 2019; Png et al., 2017). Another approach relies on surface expression of the PD-1 blocking antibody that binds interaction interface used by PD-1/PD-L1. In both cases PD-1 signaling is down due to decrease of the receptor level on cell surface or inability to bind its ligand. Despite the obvious positive effect on T cell activation caused by disruption of PD-1/PD-L1 interaction, there is limited information concerning the influence of PD-1/PD-L1 inhibition on the phenotype, cytotoxicity, and survival of therapeutic CAR-T cells. To fill this gap, we generated CD19 CAR-T cells with decreased PD-1 signaling using ER/Golgi retention by PEBL peptides or surface expression of blocking VHH specific to PD-1. We demonstrate that these modified, PD-1^low^ CD-19 CAR-T cells are highly activated and cytotoxic in comparison to control CD19 CAR-T cells but have limited therapeutic potential due to increased rate of functional exhaustion and cell death. We suggest that PD-1 signaling in addition to control of the CAR-T cell activation has positive effect on survival and persistence of functional CAR-T cells according to *in vitro* tests.

## Materials and Methods

### Expression and Purification of Biotinylated Anti-PD1 Nanobody

The nucleotide sequence coding nanobody 102c3 (ATG​GGC​TCT​AGA​GAG​GTG​CAG​CTG​GTG​GAG​TCT​GGG​GGA​GGA​TTG​GTG​CAG​GCT​GGG​AAA​TCT​CTG​AGA​CTC​TCC​TGT​GCA​GCC​TCT​GGA​AGC​ATC​TTC​AGT​ATC​CAT​GCC​ATG​GGC​TGG​TTC​CGC​CAG​GCT​CCA​GGG​AAG​GAG​CGT​GAG​TTT​GTA​GCA​GCC​ATT​ACG​TGG​AGT​GGT​GGT​ATT​ACA​TAT​TAT​GAA​GAC​TCC​GTG​AAG​GGC​CGA​TTC​ACC​ATC​TCC​AGA​GAC​AAT​GCC​AAG​AAC​ACG​GTG​TAT​CTG​CAA​ATG​AAC​AGC​CTG​AAA​CCT​GAG​GAC​ACG​GCC​ATT​TAT​TAC​TGT​GCA​GCA​GAC​CGC​GCG​GAA​TCC​TCT​TGG​TAT​GAC​TAC​TGG​GGC​CAG​GGG​ACC​CAG​GTC​ACC​GTC​TCC​TCA​GAA​CCC​AAG​ACA​CCA​AAA​CCA​CAA​CCG​GAT​CCG​TCT​CCG​T), which binds to PD1 and blocks its interaction with PD-L1 was synthesized *de novo* by Integrated DNA Technologies and cloned into the XbaI and BamHI digested pET-BAD expression vector in-frame with the 3′-terminal 6xHis tag and biotinylation signal. The resulting plasmid was transformed into BL21DE3-BirA *E.coli* strain expressing biotin ligase BirA. Individual clones were expanded in liquid medium supplemented with 0.2 mM D-biotin to produce soluble biotinylated nanobody in accordance with the protocol (Studier, 2005). Soluble 102c3 nanobody was isolated from harvested bacteria by extraction of periplasm with a buffer consisting of 50 mM Tris-HCl (pH 7.4), 150 mM NaCl, 0.1% Triton X-100 and 10 mg/ml lysozyme supplemented with PMSF followed by purification on HIS Mag Sepharose Excel (Cytiva) and dialysis against PBS pH 7.2.

### Competitive ELISA

The ability of 102c3 nanobody to block PD1/PD-L1 binding was tested by competitive ELISA. Nunc Maxisorp plates were coated with 2 μg/ml of recombinant PD-L1 and blocked with SuperBlock (Thermo). Then mixtures of PD1-Fc (2 μg/ml) and 102c3 nanobody in various molar ratios were added to plate and incubated for 1 h. After intensive rinsing detection was performed using goat anti-human IgG-HRP (sc-2453, Santa Cruz) and TMB substrate solution (Sigma-Aldrich).

### Biolayer Interferometry

To determine KD, the 102c3 nanobody was immobilized on the SA2 biosensors, which were then dipped in a series of solutions with various concentrations of PD1-Fc, and the association and dissociation rates were measured using BLItz system (ForteBio).

### Plasmid Construction

The DNA fragments coding for the third generation chimeric antigen T-cell receptor, PD-1, PD-L1, anti-PD1 nanobody 102c3 (VHH) fusions with transmembrane PDGFR containing IgG1 Fc spacer domain with modified PELLGG and ISR motifs or flexible short hinge (G_4_S)_3_ and T2a-VHH-PEBL were synthesized (GeneCust) and cloned into the pLV2 lentiviral vector (Clontech) under the control of the EF1a promoter. The arrangement of CAR genes from the N to C-terminus was as listed below: interleukin 2 signal peptide, CD19-specific ScFv (FMC63), CD8 spacer and transmembrane region, intracellular domains of the CD28, 4-1BB and CD3zetta (CAR19). CAR19-T2a-VHH-PEBL construction was generated by overlapping PCR. HEK293T cells were co-transfected with one of the viral plasmids and the set of packaging third generation plasmids (Addgene). Supernatants containing viruses were collected at 48 h post transfection.

### Cell Culture and Viral Transduction of Jurkat and Target Cells

The Nalm-6 cell line was obtained from the Institute of Cytology RAS culture collection (St. Petersburg, Russia), the HEK293T lentiviral packaging cell line was obtained from the Clontech. Jurkat-Lucia™ NFAT cells were derived from the human T cell line Jurkat by stable integration of an NFAT-inducible Lucia reporter construct (InvivoGen) encoding secreted form of luciferase. HEK293T cells were cultured in DMEM, while RPMI 1640 was used for Nalm-6, Jurkat-Lucia™ NFAT and human T-cells. Medias were supplemented with 10% FBS (Gibco), 10 mM HEPES, 100 U/ml penicillin, 100 ug/ml streptomycin, and 2 mM GlutaMAX (Gibco). Mycoplasma contamination was excluded due to regular testing of cultures by MycoReport Mycoplasma Detection Kit (Evrogen, Russia). Cell viability was calculated via trypan blue exclusion on TC20 counter (Bio-Rad). Cell transduction was performed using spinfection. Briefly, cells were re-suspended at 1 × 10^6^/ml density in 1 ml of fresh media and 1 ml of lentiviral supernatant supplemented with 5 μg/ml of Polybrene (final concentration, Millipore-Sigma). Suspensions were spun at 1,200 g for 90 min at 32°C and incubated for another 8 h at 37°C prior to replacement of viral sups by fresh media.

### Confocal Microscopy

Cells were placed on poly-D-lysin coated glasses and fixed with 4% paraformaldehyde in PBS, washed with PBS, permeabilized in 0.1% Triton X-100/PBS/0.1% FBS for 30 min at 4°C, and blocked with PBS/1% FBS/0.1% Tween-20 (PFT). To identify CAR19 distribution cells were stained with CD19 CAR Detection Reagent (1:600 in PFT) for 12 h, then samples were washed with PFT and treated for 1 h with anti-biotin-PE (Thermo Fisher Scientific, 1:1000). To assess PD1 expression, samples were treated for 12 h with anti-PD1 antibodies, conjugated with FITC (1:500 in PFT). For nuclei counterstaining 10 µM Hoechst 33,342 was used. Images were captured on an Eclipse Ti-E microscope with the A1 confocal module (Nikon Corporation) and Apo TIRF Plan Fluor 63 × 1.49.

### Luciferase Test

Jurkat-Lucia™ NFAT (Jurkat) cells were used for the luciferase tests. Target cells (Nalm-6 or Nalm-6-PD-L1 at 10^5^ cells per well of 96-well plate) were mixed with 5 × 10^4^ of either control Jurkat cells, Jurkat with PD-1 overexpression, Jurkat which express CAR19 or CAR19-T2A-VHH-PEBL only or in combination with PD-1 overexpression. After 24 h, the supernatants were collected, and the measurements were carried out according to the manufacturer’s protocol using QUANTI-Luc™ as a substrate.

### VHH 102c3 Reporter Assay

Jurkat-Lucia™ NFAT PD-1 reporter cells (5 × 10^4^) which express CAR19 and Nalm-6 PD-L1 (1 × 10^5^) were cocultured in 96-well flat bottom plates for 24 h in the presence or absence of 102c3 VHH anti-PD-1 antibody at 200, 40, 8, and 1.6 ng/ml (five-fold dilution steps). Subsequently, reporter luciferase expression was analyzed as previously described.

### T-Cell Isolation, Activation, Expansion, and Transduction

Human peripheral blood mononuclear cells (PBMCs) were isolated from the blood of healthy donors by gradient density centrifugation on a Ficoll-Paque (GE Healthcare) according to standard protocol. Peripheral blood of patients and healthy donors was obtained from Dmitry Rogachev National Research Center of Pediatric Hematology, Oncology and Immunology. All healthy donors provided informed consent. Dynabeads Untouched Human T-cells Isolation Kit (Invitrogen) was used for isolation of T cells from PBMCs. T cells were activated with CD3/CD28 Dynabeads (Thermo Scientific) at a 1:1 ratio in a media containing 40 IU/ml recombinant IL-2 (Pan Biotech) for 24 h. Activated T cells were transduced using lentiviral viral sups (CAR19 or CAR19-T2A-VHH-PEBL) according to protocol presented above for Jurkat cells. Transduced cells were grown in flasks at a density of 1.0×10^6^/ml, culture medium was replaced every 2 days.

### Sequential Killing

For prolonged cytotoxity experiment 1 × 10^4^ CAR19-T, CAR19-T2A-VHH-PEBL or control T cells were mixed with 4 × 10^4^ Nalm-6 GFP or Nalm-6 PD-L1 GFP target cells in 200 μl of complete RPMI 1640 media. Every 72 h to assess the number of CAR-T cells and target cells per well, flow cytometry analysis was made. Nalm-6 GFP or Nalm-6 PD-L1 GFP cultured without T cells were used as a negative control. Further, 15 × 10^4^ CAR-T cells from the previous round of co-cultivation were mixed with the fresh 40 × 10^4^ target cells and again incubated for 3 days. This procedure was repeated until complete disappearance of killing activity. On the day 9 portion of the T cells was used for staining of CD62L, CD45Ra, and TIGIT.

### Flow Cytometry Analysis and FACS-Based Sorting

The antibodies against the following markers were used in this study (all anti-human): CD3 APC/Cy7 (Biolegend), CD19 PE (Biolegend), PD-1 (CD279) PE (BD Biosciences) and FITC (Biolegend), PD-L1 (CD274) APC (Biolegend), CD45RA PE (Biolegend), CD62L APC (Biolegend), TIGIT PE/Cy7 (Biolegend). The CAR molecules were detected by staining with CD19 CAR Detection Reagent (Biotin) (Miltenyi Biotec), or FITC-Labeled Human CD19 (Acro Biosystems), for primary staining and streptavidin-APC/Cy7 or FITC (Abcam) for visualization. The PD1-VHH-Fc-PDGFR molecules were detected using goat cross-absorbed anti-human IgG antibody conjugated with DyLight650 (Thermo Fisher Scientific). Flow cytometric analysis was performed using NovoCyte 2060 (ACEA Biosciences) instrument, data were analyzed with FlowJo X10 (FlowJo) and NovoExpress Software (ACEA Biosciences). Jurkat-Lucia™ NFAT and Nalm-6 were transduced with the pLV_2_ PD-1 and pLV_2_ PD-L1 lentiviruses, respectively, and positive cells were sorted using SH800 (Sony) instrument.

### Statistical Analysis

Statistical processing of the experimental results was performed using the Prism 8 software package (GraphPad Software).

## Results

### Expression, Purification, and Characterization of Anti-PD1 Nanobody (VHH)

**FIGURE 1:**
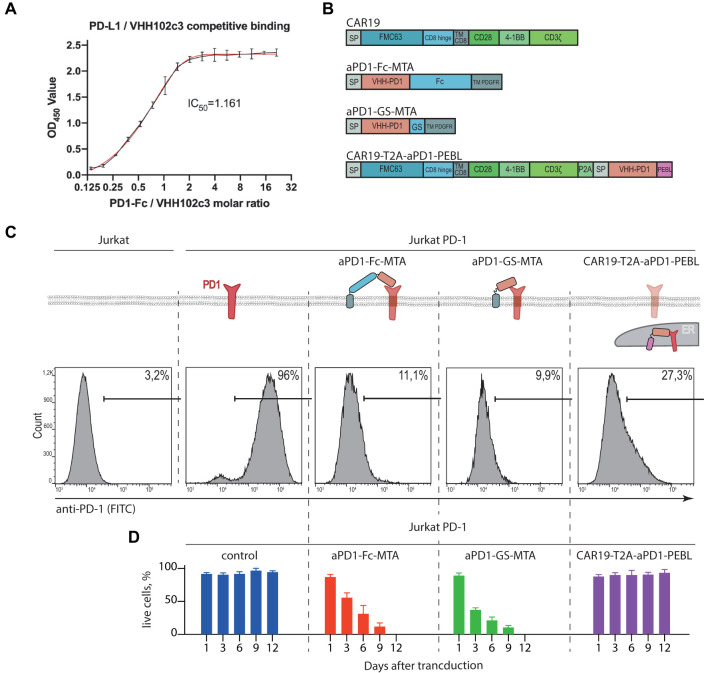
Design and characterization of the PD-1 surface expression blocking constructs. **(A)** Competitive ELISA demonstrates blocking of PD1/PD-L1 binding by 102c3 nanobody. **(B)** Scheme of lentiviral constructs designed for CAR and PD-1 stable expression, where FMC63 is scFv of anti-CD19 antibody, GS, Gly-Ser linker; TM, transmembrane domain, 4-1BB, cytoplasmic activation domain from CD137; CD3ζ, the cytoplasmic activation domain of CD3 zeta; Fc, CH2-CH3 domains of human IgG1; PDGFR, membrane anchor domain; P2A, self-cleavage peptide sequence. **(C)** Surface expression analysis of the PD-1 by Jurkat PD-1 cells after transduction by the lentiviral constructs from **(B)**. On top: diagram shows localization of the PD-1 and VHH variants anchored on cell membrane or trapped in Golgi/ER. Bottom: 48 h after transduction cells were labeled with anti-PD-1 antibodies and analyzed by flow cytometry. **(D)** Percentage of live Jurkat PD-1 cells transduced by the PD-1 surface expression blocking constructs. The number of live cells were analyzed by cell counter following trypan blue staining.

The nucleotide sequence coding for single-domain antibody 102c3 (VHH) which binds to PD1 and blocks its interaction with PD-L1 was synthesized and cloned into the pET-BAD expression vector. Nanobody 102c3 was produced using the bacterial expression system and purified. Competitive ELISA confirmed that 102c3 nanobody effectively blocks the PD1/PD-L1 interaction (IC_50_ = 1.161) (Figure 1A). To determine KD, the 102c3 nanobody was immobilized on the SA2 biosensors, which were then dipped in solutions with serial dilutions of PD1-Fc, and the association and dissociation rates were measured using BLItz system. The KD measurement demonstrates that 102c3 nanobody interacts with PD-1 in a nanomolar range (Supplementary Figure S1).

### PD-1 Downmodulation in Jurkat T Cells Using Membrane-Bound Anti-PD1 VHH or Anti-PD-1 VHH Fused to Protein Expression Blocker Sequence

To study effects of PD-1/PD-L1 signaling on the CAR-T cell activity and survival, we have chosen classic CD19 specific CAR19 (scFv clone FMC63) which can be effectively expressed in human T cells using lentiviral vector transduction. Then we designed and generated recombinant vectors expressing CD19-CAR alone, anti-PD-1 blocking VHH fused to flexible linkers (aPD-1-Fc-MTA or aPD-1-GS-MTA, Fc or (GS)_3_ linkers, respectively) followed by the transmembrane domain of PDGFR. In addition, we generated construct coding for CAR19 separated from aPD-1-VHH-PEBL (protein expression blocker, PEBL) sequence by T2A peptide (CAR19-T2A-aPD1-PEBL) for simultaneous translation (Figure 1B). Structure of resulting constructs, corresponding VHH fusion proteins, their binding to targets and expected subcellular localization are shown in Figure 1C. We expected that in the presence of aPD-1 VHH PD-1/PD-L1 interaction will be disrupted due to physical separation of these molecules or blocking of molecular interface they use for binding.

First, we stably introduced PD-1 expression cassette into human T reporter cell line Jurkat-Lucia™ NFAT Cells (Figure 1C). The ability of anti-PD-1 VHH fusions to remove ectopically expressed PD-1 from the surface of Jurkat-PD-1 T cells was tested in by immunostaining of Jurkat T cells expressing corresponding sets of recombinant proteins with anti-PD-1 mAbs conjugated to fluorophore followed by flow cytometry. On can see that in comparison to control Jurkat, Jurkat-PD-1 cells have high level of surface PD-1. Introduction of either surface-bound or fused to PEBL motif anti-PD-1 VHHs results in dramatic decrease of the surface PD-1 staining (Figure 1C). Unfortunately, we cannot rule out that, at least in part, anti-PD-1 staining could be blocked by PD-1 masking caused by interaction with anti-PD-1 VHH. But this explanation is highly unlikely since we observed very significant loss of PD-1 signal from cell surface. Surprisingly, co-expression of surface-bound anti-PD-1 VHH with both Fc and GS linkers resulted in poor survival of lentivirus-transduced cells (Figure 1D).

This fact could be possibly explained by both loss of PD-1 signaling that could protect activated T cells from apoptosis. Another explanation is additional negative co-stimulation signaling provided by the binding of VHH to PD-1. Poor survival of these double transduced Jurkat cells forced us to focus on Jurkat modified with CAR19-T2A-aPD-1-PEBL lentivirus to study consequences of PD-1 withdrawal from cell surface. In our hands this cell line showed better survival than cell lines transduced with two lentiviral constructs (Figure 1D). This observation may be explained by incomplete loss of surface PD-1; 27% of PD-1-positive cells according to staining (Figure 1C). It is possible that at least part of PEBL-anchored PD-1 may return to plasma membrane from ER/Golgi.

### Jurkat Cells Transduced With CD19 CAR-T2A- aPD-1-PEBL Construct Show Concurrent Increase in CAR Expression and Decrease in PD-1 Surface Level

**FIGURE 2:**
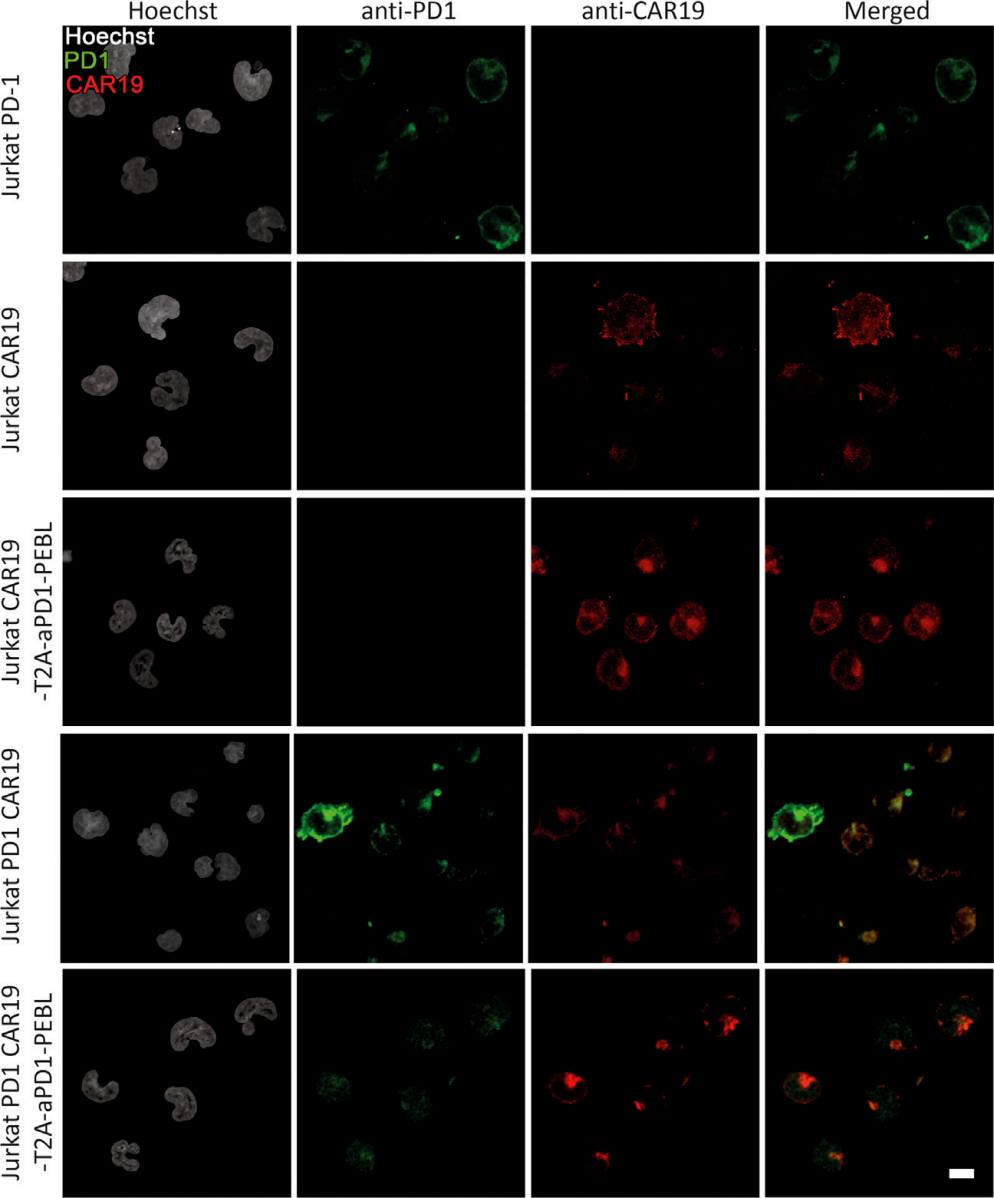
The confocal microscopy of the Jurkat and Jurkat PD-1 modified by CAR19 or CAR19-T2A-aPD-1-PEBL constructs. Jurkat and Jurkat PD-1 cells were transduced by CAR19 or CAR19-T2A-aPD-1-PEBL lentiviruses and sorted by FACS. The cells were fixed and stained with Hoechst, anti-PD-1 antibodies and CAR19 detection agent.

**FIGURE 3:**
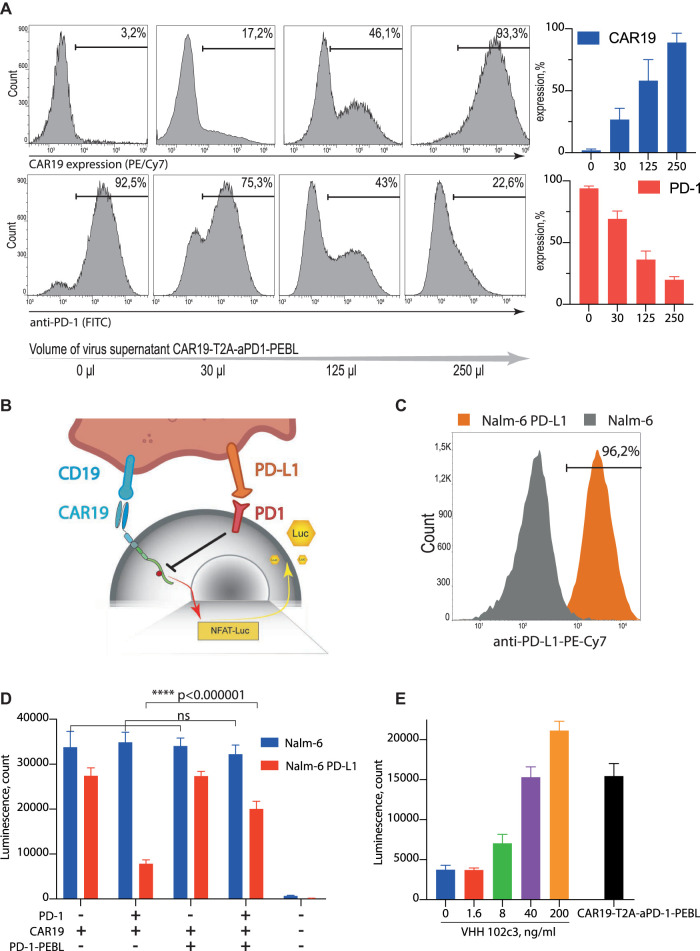
CAR19-T2A-aPD-1-PEBL construct effectively removes PD-1 from the T cells surface and releases PD-L1-mediated suppression. **(A)** Jurkat PD-1 cells were transduced by the different amounts of the CAR19-T2A-aPD-1-PEBL lentiviral supernatant and analyzed for PD-1 and CAR19 expression by flow cytometry. **(B)** Schematic representation of the Jurkat-Lucia™ NFAT PD-1 CAR19 reporter system. **(C)** Surface expression analysis of the PD-L1 on Nalm-6 and Nalm-6 PD-L1 cells. **(D)** CAR19 T cells activation luciferase assay. PD-1 positive and negative Jurkat-Lucia™ NFAT cells were modified by CAR19 or CAR19-T2A-aPD-1-PEBL and then co-incubated with Nalm-6 and Nalm-6 PD-L1. 48 h after co-incubation started, supernatant was collected and analyzed for luciferase activity. **(E)** Inhibitory effect of purified 102c3 VHH antibody on PD-L1 mediated suppression of Jurkat PD-1 CAR19 cells. Reporter Jurkat PD-1 CAR19 cells were cocultured with the Nalm-6 PD-L1 cells in presence of different concentrations of the 102c3 VHH. All data represent mean ± SD. The *p* values were determined by multiple *t* test. Statistical significance: wf****p < 0.000001.

To further investigate the effect of the PEBL construct on the PD-1 surface expression we performed the confocal imaging of the PD-1 expressing T cells modified by CAR19 or CAR19-T2A-aPD-1-PEBL constructs and found that a-PD-1-PEBL decreases the surface exposure of the PD-1 (Figure 2). We also demonstrated that transduction of Jurkat with CAR19-T2A-aPD-1-PEBL construct leads to a dose-dependent changes in CAR and PD-1 level. We used different amounts of retroviral supernatant and found that increment in CAR19 expression on the surface of transduced cells is ccompanied by decrease in PD-1 level (Figure 3A). This indicates that majority of cells expressing CAR indeed have low level of PD-1 on the surface.

### Withdrawal of PD-1 From CD19 CAR-T’s Surface Augments Their Activation in the Presence of Target Cells

Since PD-1/PD-L1 interaction provides negative signal mediated by protein phosphatase SHP-1, we expected that signaling downstream of the CAR will be diminished. This should result in decreased activity of NFAT promotor controlled by NF-kB (Figure 3B). Quantitation of the NF-AT promoter activity can be achieved by generation of CAR-T cells which carry reporter construct expressing secreted variant of firefly luciferase under control of the NFAT promoter. The reporter Jurkat-Luc and Jurkat-Luc-PD-1 cells were transduced by CAR19 or CAR19-T2A-aPD-1-PEBL lentiviruses. Next, resulting Jurkat reporter cell lines were incubated with the target B cell lymphoma cell line Nalm-6 which overexpresses PD-L1 or unmodified wild type Nalm-6 (Figure 3C). Aliquots of culture media were collected following incubation, and luciferase activity was measured. Overexpression of both PD-1 and PD-L1 in Jurkat CAR19 and Nalm-6 target cells, respectively, resulted in dramatic loss of luciferase activity in comparison to controls (low PD-1 vs. low PD-L1, or high PD-1 vs. low PD-L1) (Figure 3D). In contrast, the withdrawal of PD-1 from the surface of Jurkat-CD19 CAR-T by introduction of aPD-1-PEBL rescued secretion of luciferase and, hence, the luciferase activity (Figure 3D). Results of this experiment clearly demonstrate that CD19 CAR-T cells with restricted surface expression of PD-1 secrete more luciferase than control cell line which overexpress PD-1. This finding suggests that elimination of PD-1 from cell surface leads to enhanced activation of CAR-T cells in the presence of target cells.

To confirm that observed effects are associated with PD-1 specific blocking activity of the 102c3 VHH we co-incubated reporter Jurkat PD-1 CAR19 cells with the Nalm-6 PD-L1 cells in the presence of different concentrations of the 102c3 VHH. As expected, increasing doses of 102c3 VHH rescued Jurkat PD-1 CAR-19 cells from PD-L1-mediated suppression (Figure 3E).

### aPD-1-PEBL Removes PD-1 From Cell Surface in Human Donor T Cells

**FIGURE 4:**
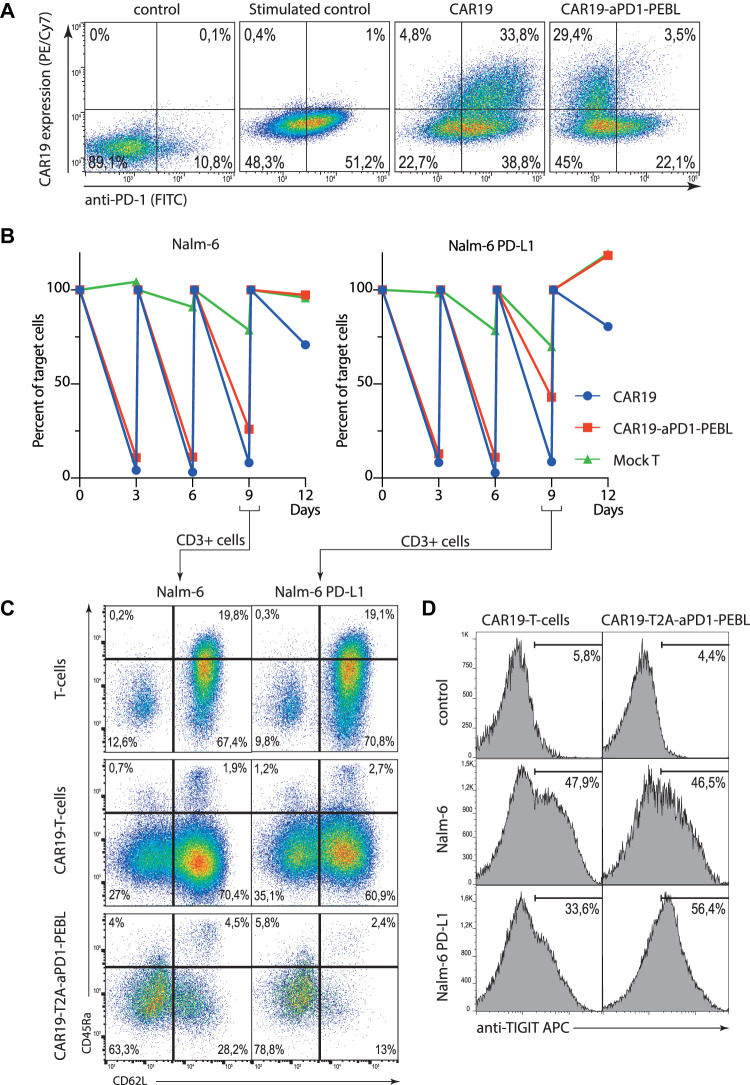
The blocking of PD-1 surface expression by the CAR19-T2A-aPD-1-PEBL in peripheral blood human T cells results in increased activation and exhaustion. **(A)** Transduction efficacy of activated, CD3/CD28 bead-expanded human T cells with lentiviral-based vectors expressing CAR19 and CAR19-T2A-aPD-1-PEBL constructs. Cells were analyzed for surface expression of PD-1 and CAR19 by flow cytometry. **(B)** Sequential killing assay of CAR19 or CAR19-T2A-aPD-1-PEBL T cells incubated with Nalm-6 or Nalm-6 PD-L1 target cells in 1:4 ratio for 12 days. Number of target cells per well were analyzed every 3 days by flow cytometry. **(C)** Flow cytometry analysis of the phenotype of CAR19 and CAR19-T2A-aPD-1-PEBL T cells incubated with Nalm-6 or Nalm-6 PD-L1 from **(B)** on day 9. **(D)** FACS quantification of CAR19 and CAR19-T2A-aPD-1-PEBL T cells expressing exhaustion marker TIGIT among CD3^+^ cells in samples from **(B)** on day 9.

To demonstrate that aPD-1-PEBL is also efficient in locking PD-1 inside human donor T cells, we isolated human CD3-positive T cells from the peripheral blood and transduced them with CD19 CAR-T2A-aPD-1-PEBL lentiviral particles (Figure 4A). One can see that CAR-expressing cells demonstrate low level of surface PD-1 in comparison to control cells expressing CAR only. This finding has important implications for functional experiments as it will be shown below.

### PD-1 Elimination From the CAR-T Surface Impairs Their Killing Activity

As it was shown before, CAR-T cells with terminally differentiated and exhausted phenotype have decreased killing activity according to degranulation and target cell killing assays (Wherry and Kurachi, 2015; Tantalo et al., 2021). Side by side comparison of CAR19-T2A-aPD-1-PEBL and control CAR19 T cells revealed that in sequential killing tests both CAR-Ts have similar killing activity at early time points when they see target cells for the first time (Figure 4B). One can notice that CAR19 T cells effectively eliminate both Nalm-6 and Nalm-6 PD-L1 cells in sequential killing assay (Figure 4B). This result is puzzling since CAR-T cells usually upregulate PD-1 following stimulation, and this was the case in our experiment. But according to flow cytometry the PD-1 expression on the activated T cells gradually decreases being the highest on day 1 and almost undetectable at day 8 (Supplementary Figure S2). But eventually when fresh target cells are added, CD19 CAR-T aPD-1-PEBL T cells rapidly lose their killing capacity in comparison to control CAR-Ts with high level of PD-1 (Figure 4B). This result proves that for prolonged CAR-T activity and successful target cell killing the strength of activation signal provided by CAR should be balanced by negative signal from immune control points such as PD-1. It also demonstrates that simple elimination/knockdown of PD-1 is not the best option if the one would like to optimize CAR-T cells by disposing of negative co-stimulation.

### CD19 CAR-T Cells With Low PD-1 Have Higher Proportions of Terminally Differentiated and Exhausted Cells

As it was mentioned before, the removal of PD-1 from the cell surface leads to poor survival of CAR19 T cells. To figure out the reason behind this unwanted effect, we studied expression of differentiation markers as well as markers characteristic for T cell exhaustion in CAR-Ts with high and low levels of PD-1. Surface phenotype of CAR-T cells was assessed by direct immunostaining of control, CAR19 or CAR19-T2A-aPD-1-PEBL T cells with antibodies against CD62L and CD45RA in the course of Nalm-6 or Nalm-6 PD-L1 killing assay. The results of this experiment demonstrate that stimulation of CAR-T cells with low level of PD-1 leads to increased fractions of terminally differentiated cells (Figure 3C). CAR19-T2A-aPD-1-PEBL T cells had the highest fraction of effector memory (EM) T cells in comparison to control CD19 CAR-Ts (Figure 4C). In addition to accumulation of EM cells, CAR19-T2A-aPD-1-PEBL T cells had increased proportion of TIGIT-positive cells (Figure 4D). TIGIT (T cell immunoreceptor with Ig and ITIM domains) expression is upregulated in tumor infiltrating CD8 lymphocytes with exhausted phenotype according to reduced immune signaling (Thommen and Schumacher, 2018; Liu et al., 2020; Simon et al., 2020; Ostroumov et al., 2021). This molecule is also associated with exhausted dysfunctional phenotype of human CAR-T cells, and like PD-1 is considered as critical negative regulator of T cell activation. We concluded that PD-1 elimination from the surface of CD19 CAR-T causes increased activation, faster maturation, and functional exhaustion of T cells. Perhaps, CAR-T cells are more sensitive to stimulation associated apoptosis in the absence of PD-1 signal which normally attenuates strong signal through CAR. Thus, paradoxically, decreased negative signaling led to worse survival, and dysfunction of CAR-T cells, at least in our *in vitro* study.

## Discussion

Immune suppressive tumor microenvironment provides a major challenge for development of the new therapeutic approaches based on adoptive transfer of donor tumor specific T cells. It holds true for CAR-T therapy as well. CD19 CAR-T cells used for treatment of B cell lymphomas and leukemias upregulate suppressive receptors such as CTLA-4 and PD-1 which limit their activation and cytotoxicity. PD-1 expression is induced by elevated level of its ligand PD-L1 which rapidly increases in tumor cells. Negative signaling provided by PD-1 results in activation of protein phosphatases that dephosphorylate ITAM motifs involved in T cell stimulation and effector function such as cytotoxicity. Therefore, the idea to disrupt PD-1/PD-L1 interaction and break negative co-stimulation in CAR-T cells is very engaging. One can knockout PD-1 gene, downregulate PD-1 transcription level, target PD-1 mRNA for degradation, affect protein stability, and break ability of PD-1 to bind PD-L1 by using monoclonal antibodies to relocate PD-1 inside the cell or block the binding interface of PD-1. The approaches relying on protein engineering of surface proteins are very promising since they directly affect signaling events without touching transcriptional regulation. In our study we used VHH parts of nanobodies against PD-1 expressed on the surface of CD19 CAR-T cells to block PD-L1 binding and induce loss of surface expression of PD-1. Alternatively, we co-expressed PD-1, CD19 CAR and aPD-1 VHH fused to PEBL motif. It was demonstrated earlier that short PEBL peptide that we used in this work is sufficient to relocate and lock proteins, in particular PD-1, inside Golgi/ER. According to our expectations, removal of PD-1 from cell surface would result in stronger CAR signaling and more efficient killing of target cells. To our great surprise, we evidenced decreased survival of CAR-T cells, rapid terminal differentiation and functional exhaustion of CAR-T cells with low level of PD-1. This finding matches other’s data showing that inhibition of PD-1 signaling may negatively affect survival and functional fitness of CAR-T cells (Wei et al., 2019). In short term experiments, CAR-T cells with low level of PD-1 on cell surface have comparable ability to kill target cells, but sequential killing experiments revealed that removal of PD-1 and, hence, loss of PD-1 signaling has negative effect on survival and/or cytotoxicity of CAR-T cells. We speculate that PD-1 may attenuate excessive stimulus provided by CAR which, in the absence of breaks, increases proliferation induced apoptosis.

In conclusion, it should be noted that for the first time we demonstrated that VHH domain of 102c3 nanobody against PD-1 can be used for efficient blocking of PD-1/PD-L1 interaction in Jurkat or human donor T cells. This domain binds PD-1 with nanomolar KD which is sufficient for relocation of PD-1 from outer membrane to Golgi/ER using PEBL peptide fused to VHH domain. Increased signaling caused by restriction of PD-1 signaling (due to binding blockage or relocation from outer membrane) has negative effect on CD19 CAR-T ability to survive and kill target lymphoma cells. From practical standpoint, restriction of PD-1 signaling should not be used for improvement of CAR-T persistence and killing ability. Other immune control checkpoints or targets should be used to avoid negative effects of tumor microenvironment on CAR-T cells.

## Data Availability

The raw data supporting the conclusion of this article will be made available by the authors, without undue reservation.
