# Supplementary material for: Engineered Removal of PD-1 From the Surface of CD19 CAR-T Cells Results in Increased Activation and Diminished Survival
Source: Front Mol Biosci. 2021 Oct 13;8:745286. doi: 10.3389/fmolb.2021.745286 (PMC8548718; doi:10.3389/fmolb.2021.745286)
Supplement: Supplementary file 6 [file DataSheet1.docx]

## Supplementary Figures


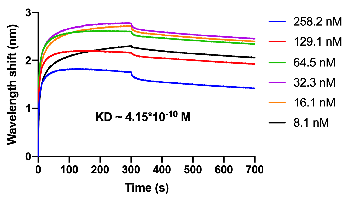


**Supplementary Figure 1. Affinity of the 102c3 nanobody binding to recombinant PD-1**. To determine KD, the 102c3 nanobody was immobilized on the SA2 biosensors, which were then dipped in a series of solutions with various concentrations of PD1-Fc, and the association and dissociation rates were measured using BLItz system (ForteBio).

**Supplementary Figure 2. Inhibition of PD-1 surface staining in dependence of concentration of the 102c3 VHH**.

**Supplementary Figure 3. Flow cytometry analysis of PD-1 expression on the surface of CAR19 T cells after activation and transduction**.
